# Increased Dendritic Spine Density and Tau Expression Are Associated with Individual Differences in Steroidal Regulation of Male Sexual Behavior

**DOI:** 10.1371/journal.pone.0069672

**Published:** 2013-07-16

**Authors:** Pranay Bharadwaj, Christine McInnis, Amanda M. K. Madden, Paul J. Bonthuis, Susan Zup, Emilie F. Rissman, Jin Ho Park

**Affiliations:** 1 Psychology Department, University of Massachusetts, Boston, Boston, Massachusetts, United States of America; 2 Department of Biology, Brandeis University, Waltham, Massachusetts, United States of America; 3 Department of Neurobiology and Anatomy, University of Utah, School of Medicine, Salt Lake City, Utah, United States of America; 4 Department of Biochemistry & Molecular Genetics, University of Virginia School of Medicine, Charlottesville, Virginia, United States of America; CNRS UMR7275, France

## Abstract

Male sexual behavior (MSB) is modulated by gonadal steroids, yet this relationship is highly variable across species and between individuals. A significant percentage (∼30%) of B6D2F1 hybrid male mice demonstrate MSB after long-term orchidectomy (herein after referred to as “maters”), providing an opportunity to examine the mechanisms that underlie individual differences in steroidal regulation of MSB. Use of gene expression arrays comparing maters and non-maters has provided a first pass look at the genetic underpinnings of steroid-independent MSB. Surprisingly, of the ∼500 genes in the medial preoptic area (MPOA) that differed between maters and non-maters, no steroid hormone or receptor genes were differentially expressed between the two groups. Interestingly, best known for their association with Alzheimer’s disease, amyloid precursor protein (*APP*) and the microtubule-associated protein tau (*MAPT*) were elevated in maters. Increased levels of their protein products (APP and tau) in their non-pathological states have been implicated in cell survival, neuroprotection, and supporting synaptic integrity. Here we tested transgenic mice that overexpress tau and found facilitated mounting and intromission behavior after long-term orchidectomy relative to littermate controls. In addition, levels of synaptophysin and spinophilin, proteins generally enriched in synapses and dendritic spines respectively, were elevated in the MPOA of maters. Dendritic morphology was also assessed in Golgi-impregnated brains of orchidectomized B6D2F1 males, and hybrid maters exhibited greater dendritic spine density in MPOA neurons. In sum, we show for the first time that retention of MSB in the absence of steroids is correlated with morphological differences in neurons.

## Introduction

Gonadal steroids modulate a myriad of male social behaviors that contribute to successful reproduction and survival. In most rodent animal models studied in the lab, the dependence of male sexual behavior (MSB) on gonadal steroids is well documented (reviewed in [1]). However, one of the defining characteristics of MSB is the high variability found among individuals, as striking inter-individual variation in the response to orchidectomy has been well documented in numerous species (reviewed in [2]). There are very few rodent models that provide the opportunity to parse out the non-steroidal mechanisms that regulate MSB [3], [4]. One of these models is the B6D2F1 hybrid mouse strain in which ∼30% of the males retain the ejaculatory reflex for as many as 25 weeks after orchidectomy (herein after referred to as maters; [5], [6]).

Hormonal characterization of this hybrid strain has revealed nothing exceptional [7], [8]. Thus, microarray gene expression analyses of the medial preoptic area (MPOA), an area integral for MSB, were done to provide a first pass look at the genetic underpinnings of steroid-independent MSB [9]. Over 500 genes were differentially expressed between maters and non-maters. Cross referencing this list with genotype data mined from B6D2F1 recombinant inbred mouse lines led to the identification of a group of genes that were associated with neurodegenerative diseases and steroid-independent MSB. Expression of two genes in particular, amyloid beta (A4) precursor protein (*APP*) and microtubule associated protein tau (*MAPT*), which are normally associated with Alzheimer’s Disease, was found to be significantly higher in the maters relative to the non-maters [9]. Furthermore, transgenic male mice that overexpressed APP displayed enhanced sexual behavior, demonstrating a causal relationship between APP and MSB [9].

To continue this work here, we determined the levels of the protein product of *MAPT* (tau) between maters and non-maters, and we tested for MSB in tau overexpressors prior to and after orchidectomy. Tau is a microtubule-associated protein which maintains the normal morphology of neurons by modulating the assembly and stabilization of microtubules [10]–[12]. Neuronal microtubules serve as “highways” for axonal transport and, by extension, are involved in supporting synaptic integrity and neuronal viability. Thus, we predicted that levels of other proteins associated with synaptic connectivity, such as synaptophysin and spinophilin, would also be elevated in maters in the MPOA. To determine whether differences in dendritic morphology were associated with steroid-independent MSB, Golgi-impregnated MPOA neurons in brains of orchidectomized B6D2F1 males were assessed. Based on the bioinformatic analyses of the microarray data which indicated that structural differences at the synaptic level may play a significant role in steroid-independent MSB [9], we predicted there would be greater structural complexity in the dendritic morphology of MPOA neurons of maters relative to that of non-maters.

## Materials and Methods

### Experiment 1: Tau, Synaptophysin, and Spinophilin Levels in Maters vs. Non-maters

#### Animals

Male B6D2F1 hybrid mice (*Mus musculus; n = 32)* were produced by crossing C57BL/6J females with DBA/2J males. All males were raised in the animal facility at the University of Massachusetts, Boston, weaned at 20–21 days, and then housed singly until the onset of the experiments (between 50 and 80 d of age). Animals were given food (Prolab RMH 3000; PMI International, Brentwood, MO) and water *ad libitum* and maintained on a 12∶12 light:dark cycle, with lights off at 1200 h EST.

All procedures for Experiments 1–3 were performed according to the AALAC guidelines and approved by the University of Virginia Animal Use and Care Committee and by the University of MA, Boston IACUC. All surgeries and sacrifices that were performed in Experiments 1–3 were performed while the animals were anesthetized with isoflurane, and all efforts were made to minimize suffering.

#### Behavioral testing

To test for MSB, males were placed into Plexiglas arenas (17.8 cm w×17.8 cm h×25.4 cm l) with their home cage bedding which had not been changed for at least 1–2 weeks, and then the males were habituated for one hour prior to stimulus female introduction. C57BL/6J females were used as stimulus females for MSB testing. Females were ovariectomized and injected subcutaneously with 5 µg estradiol benzoate (dissolved in sesame oil) 48 h prior to testing. Three to five hours prior to testing, stimulus females were injected subcutaneously with 5 µg progesterone. The females were group-housed in the same colony room as the experimental males.

All tests were conducted under dim red lights during the dark phase of the light/dark cycle. Tests began with the introduction of a hormone-treated stimulus female, and once the male mounted the test continued to a criterion of a successful ejaculatory reflex or for 120 min, whichever occurred first. If the stimulus female became unreceptive during testing she was replaced with a receptive female.

All tests were videotaped and scored by an observer blind to the classification of the individuals. During each behavioral test, the behavioral components recorded were: mount latency (ML; time from the introduction of a receptive female to the first mount), intromission latency (IL; time from the introduction of a receptive female to the first intromission), and ejaculation latency (EL; interval between the first intromission and ejaculation).

B6D2F1 hybrid males were given 4 weekly MSB tests prior to orchidectomy, and all the males ejaculated on at least 3 of the four tests. Thus, all the males were considered sexually experienced and chosen for further study. After orchidectomy, males were tested for MSB every two weeks for 16 weeks after orchidectomy. Males were considered to be “maters” if they demonstrated mounts, intromissions and the ejaculation reflex on at least three out of the last four behavioral tests, including the last test (n = 6). Males were considered non-maters (n = 8) if they did not display any of the components of MSB during the last four tests.

#### Western blot analysis

One day after the completion of the sexual behavior tests, mice were sacrificed, and brains were removed, rapidly frozen, and then stored at −80°C until they were cut into 100 µm thick coronal sections with a Leica cryostat. Based on the Franklin and Paxinos mouse brain atlas (Franklin and Paxinos, 2008), the MPOA, medial amygdala, and frontal cortex were dissected and homogenized in Thermo Scientific Tissue Protein Extraction Reagent (TPER) plus HALT protease inhibitor chilled on ice. Samples were stored at −80°C. For protein extraction, brain tissue homogenates were thawed and centrifuged, and total protein concentrations were determined by BCA (bicinchoninic acid) Protein Assays (Pierce Chemical Co., Rockford, IL). Samples were loaded into a 10% polyacrylamide gel and subjected to electrophoresis and transferred to a nitrocellulose membrane. Membranes were blocked in 10% milk in Tween TBS overnight at 4°C then warmed to room temperature and rinsed. They were then incubated with either Anti-Tau monoclonal antibody, clone 46 produced in mouse (1∶10,000; Sigma-Aldrich Corp., T9450) followed by polyclonal goat anti-mouse (1∶10,000; BioRad, 170–5047), polyclonal anti-spinophilin/neurabin II produced in rabbit (1∶1000, Sigma-Aldrich Corp., N5162) followed by polyclonal goat anti-rabbit (1∶10,000; Millipore, AP307P), or monoclonal anti-synaptophysin produced in rabbit (1∶10,000; Millipore, catalog MAB368) followed by polyclonal goat anti-rabbit (1∶10,000; Millipore, AP307P). This was followed by detection using SuperSignal® West Pico Chemiluminescent Substrate (Pierce Chemical Co.). Later, blots were reprobed with antibody against β-actin (1∶50,000; Sigma-Aldrich Corp. A1978), and after rinsing, the blots were incubated for 1 h in an HRP-conjugated goat anti-mouse IgG secondary antibody (1∶10,000; Jackson) followed by chemiluminescent detection. The intensities of each of the candidate proteins and β-actin were visualized and quantified directly using the Bio-Rad Chemi Doc XRS+ Imager and Image Lab software (BioRad, USA). Levels of each of the proteins were then normalized to those of β-actin in each sample. Manuals of the BioRad Image Lab software are available on their website (http://www.bio-rad.com/).

### Experiment 2: MSB Before and After Orchidectomy in Tau Overexpressing Mice

#### Animals

Adult (∼8 weeks old) transgenic male mice that overexpress tau (known as *htau* mice; n = 13) and littermate controls (n = 10) were purchased from the Jackson Lab (*Mapt^tm1(EGFP)Klt^* Tg(MAPT)8cPdav/J; stock # 4808). These mice were developed by crossing transgenic mice (mouse line 8C) with mice targeted to be human *MAPT* mutants. An EGFP coding sequence was inserted into the first *MAPT* exon and disrupted expression of the gene and produced a cytoplasmic EGFP protein fused to the first 31 amino acids of MAPT. The transgenic allele is a PAC insert of 200–250 kb including the coding sequence, intronic regions and regulatory elements of the human *MAPT* gene. The founders of this line have now been crossed into the C57BL/6J line for ten generations. These mice express all six human MAPT isoforms. No endogenous mouse MAPT is detectable. All of the tau overexpressing mice and littermate controls were tested in the Jordan Hall Vivarium at the University of Virginia, Charlottesville. Mice were singly housed between tests.

#### Behavioral testing and western blot analyses

After a two week acclimation period, tau overexpressors and their littermate controls were provided with 4 weekly MSB tests prior to orchidectomy. They were then tested for MSB weekly for 12 weeks after orchidectomy as detailed above. One day after the completion of the final sexual behavior test, mice were sacrificed, and their brains were dissected and prepared for Western Blot analyses for tau, synaptophysin, and spinophilin as described in Experiment 1.

### Experiment 3: Dendritic Morphology of MPOA Neurons in Maters and Non-maters

#### Animals and behavioral testing

Male B6D2F1 hybrid mice (n = 15) were provided with 4 weekly MSB tests prior to orchidectomy. All the males ejaculated on at least 3 of the four tests and were considered sexually experienced. Males were then tested weekly for MSB for 11 weeks after orchidectomy. Males were considered to be “maters” if they demonstrated the ejaculation reflex on at least two out of the last three behavioral tests, including the last test (n = 5). Males that did not display MSB during the last three tests were considered non-maters (n = 5).

#### Golgi impregnation

Maters and non-maters were perfused with 8% paraformaldehyde one day after the last behavioral test. Brains were subjected to Golgi staining using the FD Rapid GolgiStain Kit (FD NeuroTechnologies, Ellicot City, MD) according to the manufacturer’s instructions. The fixed brains were sliced on a cryostat (Leica CM3050S; Buffalo Grove, IL) at a section thickness of 150 µm. The sections were mounted onto superfrost slides (Fisherbrand). Sections were protected from light and left overnight on the slides to dry. The slides were then dehydrated and cleared with xylene as per the manufacturer’s protocols and coverslipped under Permount.

#### Morphological analyses

Slides were coded prior to data collection, and the code was broken after analyses were complete. Images of Golgi-impregnated neurons in the MPOA were captured using an Olympus DP-72 microscope equipped with a camera linked to a computer with DP2–BSW software (version 2.2, build 6212, Olympus) and Image J software (http://rsb.info.nih.gov/ij/; NIH). For each brain, MPOA neurons were selected for analyses only if they were thoroughly impregnated and sufficiently isolated from surrounding cells. Analyses for total dendritic length, number of branches, and dendritic spine density were conducted. For dendritic length and branching analyses, 5 neurons per animal were selected at random. Images were taken at 40× magnification. Golgi-impregnated cells were then traced, and an average of the total dendritic tree length per neuron was calculated. Branch points were counted when a dendrite exhibited a distinct bifurcation arising mostly from, but not limited to, the main branch. For analyses of dendritic spine density, 5 MPOA neurons that satisfied the criteria described above were randomly chosen. On each neuron, 5 dendritic segments (∼65 µm in average length) were randomly selected, and spine density was analyzed at 100× magnification. Dendritic segments were randomly chosen so that they were mostly in one focal plane. Only spines extending away from the shaft were counted.

#### Statistical analysis

Chi-square tests were used to compare differences in the proportion of males displaying copulatory behavior between groups. One-way ANOVAs were used to analyze average dendritic spine density, average total dendritic branch length, average number of branch points per neuron, and protein levels between groups. Data from one individual in the analysis of the synaptophysin protein levels and two individuals in the analysis of the spinophilin protein levels in the MPOA were considered outliers and omitted, as they were greater than at least three standard deviations from the mean. Post-hoc comparisons were conducted using the Fisher Protected Least Significant Difference test where appropriate. Observed differences were considered significant if *p*<0.05. Statistical tests were run using the Statview program (Statview 5; SAS Institute, Cary, NC, USA).

For differences in the median latencies to the first mount, intromission, and ejaculation between control and tau overexpressing mice, Kaplan-Meier curves were generated to show the percentage of mice displaying MSB at a specific time point over all behavioral trials pre- orchidectomy (4 trials×7,200 s/trial = 28800 s total), and all trials post- orchidectomy (10 trials×7,200 s/trial = 72,000 s total). Any animal that never mounted in any post- orchidectomy trial was a censored data point of 72,000 s, indicating that the animals’ true latency to mount was longer than 72,000 s. Any animal that did not mount was dropped from the data set for latency to intromit and ejaculate. The latency to intromit for an animal that mounted but did not intromit in any trial was a censored data point. This was calculated as 28,800 s for pre- orchidectomy and 72,000 s for post-orchidectomy minus the latency to mount, and the test was dropped from the data set for latency to ejaculate only. The latency to ejaculate for an animal that intromitted but did not ejaculate, was a censored data point calculated as 28,800 s for pre-orchidectomy, and 72,000 s for post-orchidectomy minus latency to intromit starting from when the female was introduced. Statistical differences between curves were analyzed by Mantel-Cox log-rank tests in GraphPad Prism 5 software.

## Results

### Experiment 1: Tau, Synaptophysin and Spinophilin are Higher in B6D2F1 Hybrid Maters vs. Non-maters

**Figure 1:**
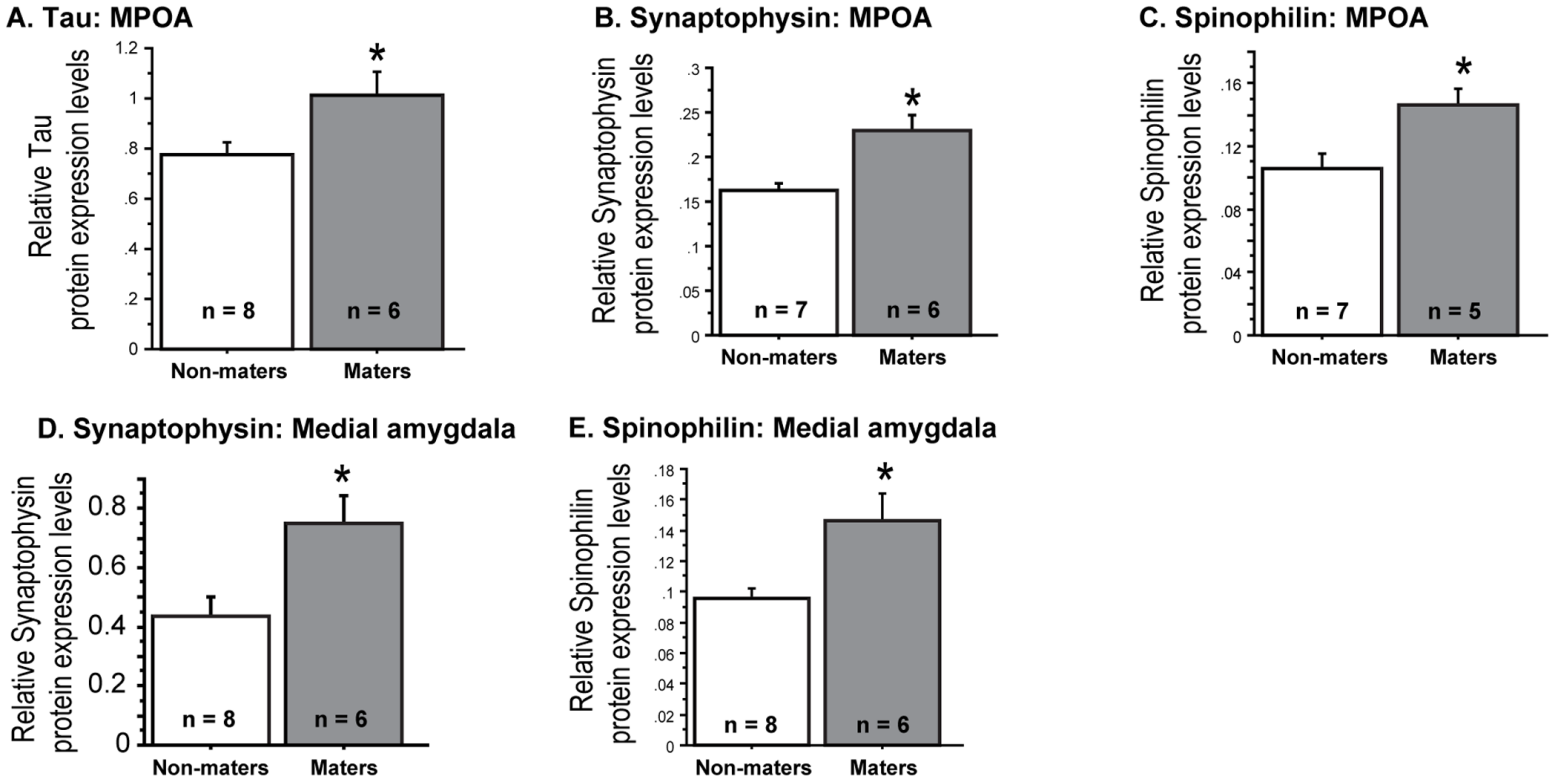
Tau, synaptophysin and spinophilin levels in the medial preoptic area in orchidectomized B6D2F1 hybrid male mice. B6D2F1 hybrid males that continued to demonstrate male sexual behavior after long-term orchidectomy (“maters”) have significantly more tau, synaptophysin and spinophilin in the MPOA (A-C) and more synaptophysin and spinophilin in the medial amygdala (D-E) than B6D2F1 hybrid males that ceased after long-term orchidectomy (“non-maters”). Between the two groups, there were no differences in tau levels in the medial amygdala or frontal cortex (data not illustrated). In addition, no differences in synaptophysin or spinophilin protein levels were observed in the frontal cortex between the two groups (data not illustrated). (*p<0.05; levels normalized with an endogenous control, β-actin).

*MAPT* is constitutively expressed in the central and peripheral nervous systems [13]. In order to assess the validity of data generated from a previously conducted gene expression array [9], western blots of the tau protein from MPOA tissues of orchidectomized B6D2F1 hybrid male mice were conducted. The results showed that tau was ∼24% higher in maters than non-maters (F_(2,19)_ = 2.812, p<0.05; Figure 1A). Since elevated tau was only observed in the MPOA, and not the medial amygdala or frontal cortex (*p*>0.05 in all comparisons; data not illustrated), steroid-independent MSB in maters may be attributed to a very regional increase in tau.

Synaptophysin is an essential transmembrane glycoprotein in presynaptic vesicles involved in structural functions and may play a significant role in the regulation of synaptic plasticity ([14]; reviewed in [15]). Spinophilin is implicated in regulating spine formation and function, and the actin-binding domain of spinophilin is both necessary and sufficient for targeting of spinophilin to dendrites and dendritic spines [16]–[19]. Because both APP and tau have been implicated in supporting synaptic integrity, we predicted that the levels of synaptophysin and spinophilin would also be elevated in the hybrid maters. Both synaptophysin and spinophilin protein levels in the MPOA were higher in maters relative to non-maters (F_(2,18)_ = 5.315, p<0.05 and F_(2,17)_ = 3.950, p<0.05, respectively; Figure 1B and 1C). Interestingly, unlike tau, synaptophysin and spinophilin levels in the medial amygdala of hybrid maters were significantly higher than those observed in the non-maters (F_(2,19)_ = 4.444, p<0.05 and F_(2,19)_ = 2.84, p<0.05, respectively; Figure 1D-E). In addition to the MPOA, the medial amygdala is an important integration site underlying MSB [1], [20]. No differences in synaptophysin or spinophilin protein levels were observed in the frontal cortex between the two groups (*p*>0.05 in all comparisons; data not illustrated).

### Experiment 2: Mice Overexpressing Tau Demonstrate Facilitated Mounting and Intromission Behavior After Orchidectomy

**Figure 2:**
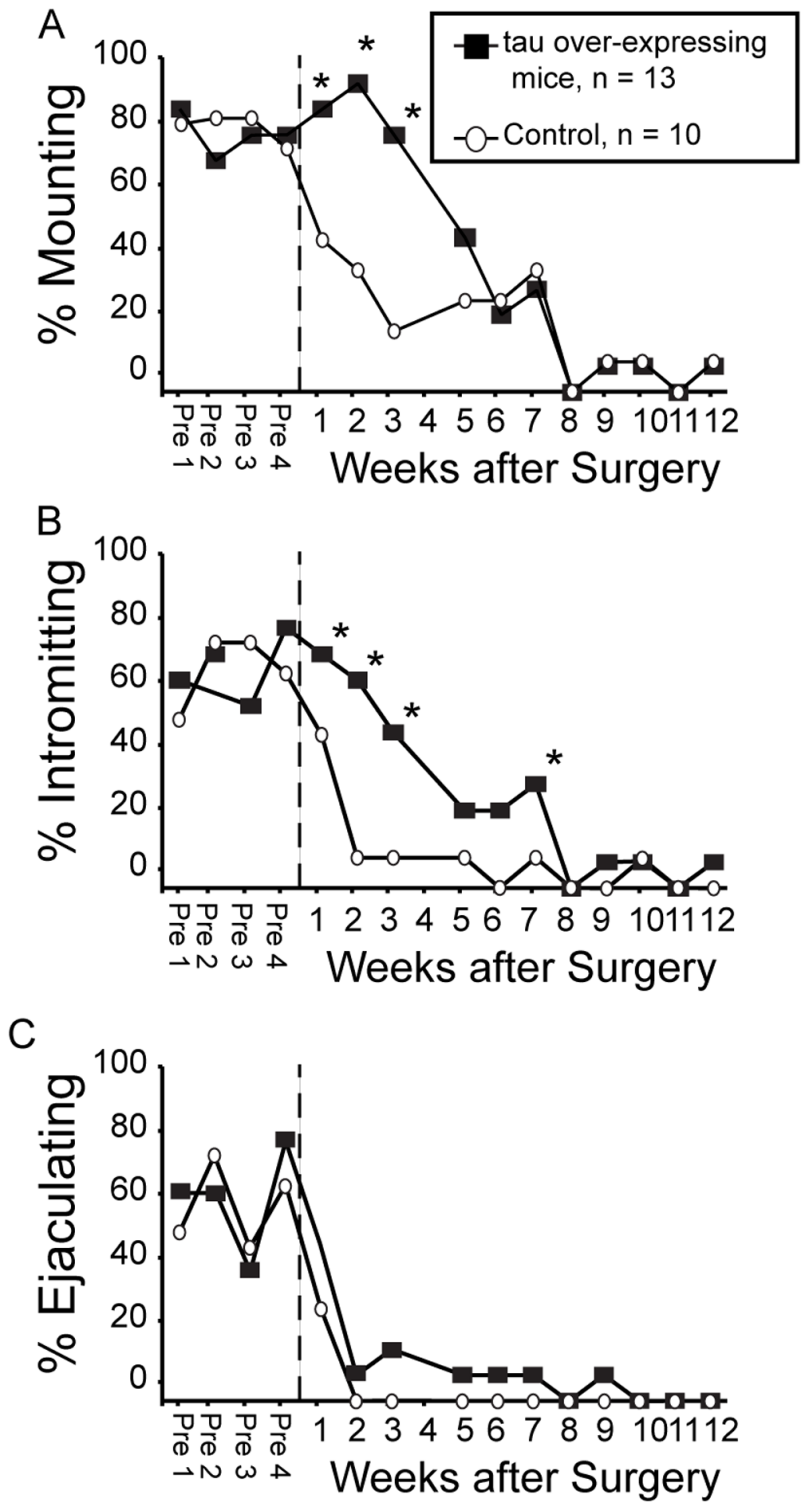
Sexual behavior in tau overexpressing mice and littermate controls. Percentage of mice that displayed (A) mounting, (B) intromissions, and (C) an ejaculatory reflex prior to and after orchidectomy. *Significantly higher than littermate controls (p<0.05).

**Figure 3:**
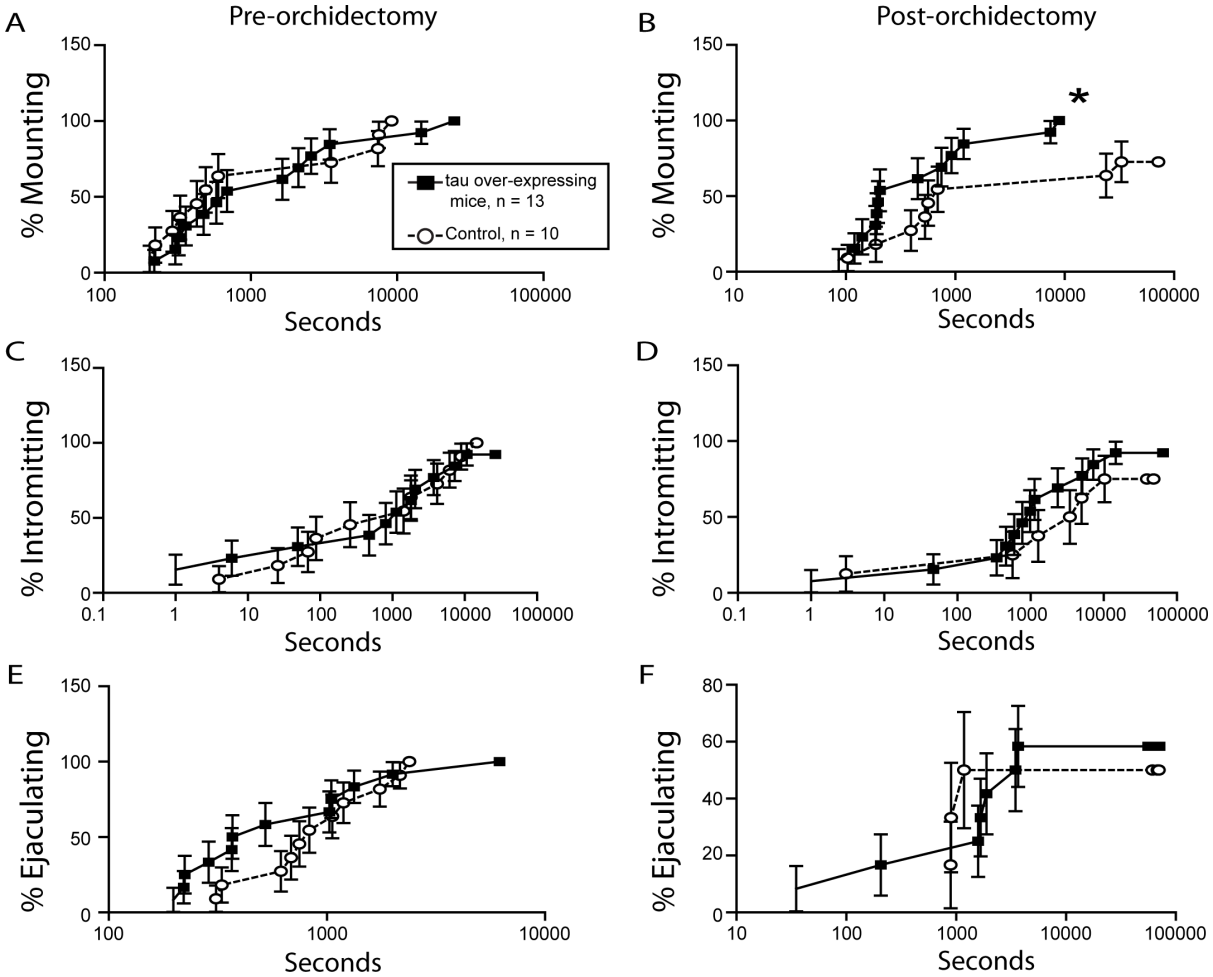
Kaplan-Meyer survivability plots of male sexual behavior of tau overexpressing mice. Kaplan-Meyer survivability plots for the percent accumulation of tau overexpressing mice reaching their first (A-B) mount, (C-D) intromission, and (E-F) ejaculation over time in seconds prior to orchidectomy and after orchidectomy. *Significantly different from littermate controls (p<0.05).

Because relative expression of the *MAPT* gene was increased in the B6D2F1 hybrid maters compared to non-maters, we hypothesized that gonadal steroid-independent MSB would be facilitated in tau overexpressing mice when compared with their littermate controls. From weeks 1–3 after orchidectomy, a larger percentage of tau overexpressors displayed increased mounts when compared to littermate controls (p<0.05; Figure 2A). A higher percentage of tau overexpressing mice demonstrated intromissions when compared to littermate controls up to 7 weeks after orchidectomy (p<0.05; Figure 2B). One tau overexpressor demonstrated the ejaculatory reflex 7 and 9 weeks after orchidectomy; however, there was not a statistical difference in the percentage of males that demonstrated the ejaculatory reflex between groups (Figure 2C). In addition, latencies to mount for tau overexpressing mice were significantly different from littermate controls after orchidectomy, as revealed by the survival curves generated from the Mantel-Cox log-rank test (p<0.05; Figure 3B). Latencies to intromit or ejaculate were not significantly different between the two groups after orchidectomy (p>0.05; Figures 3D & 3F).

**Figure 4:**
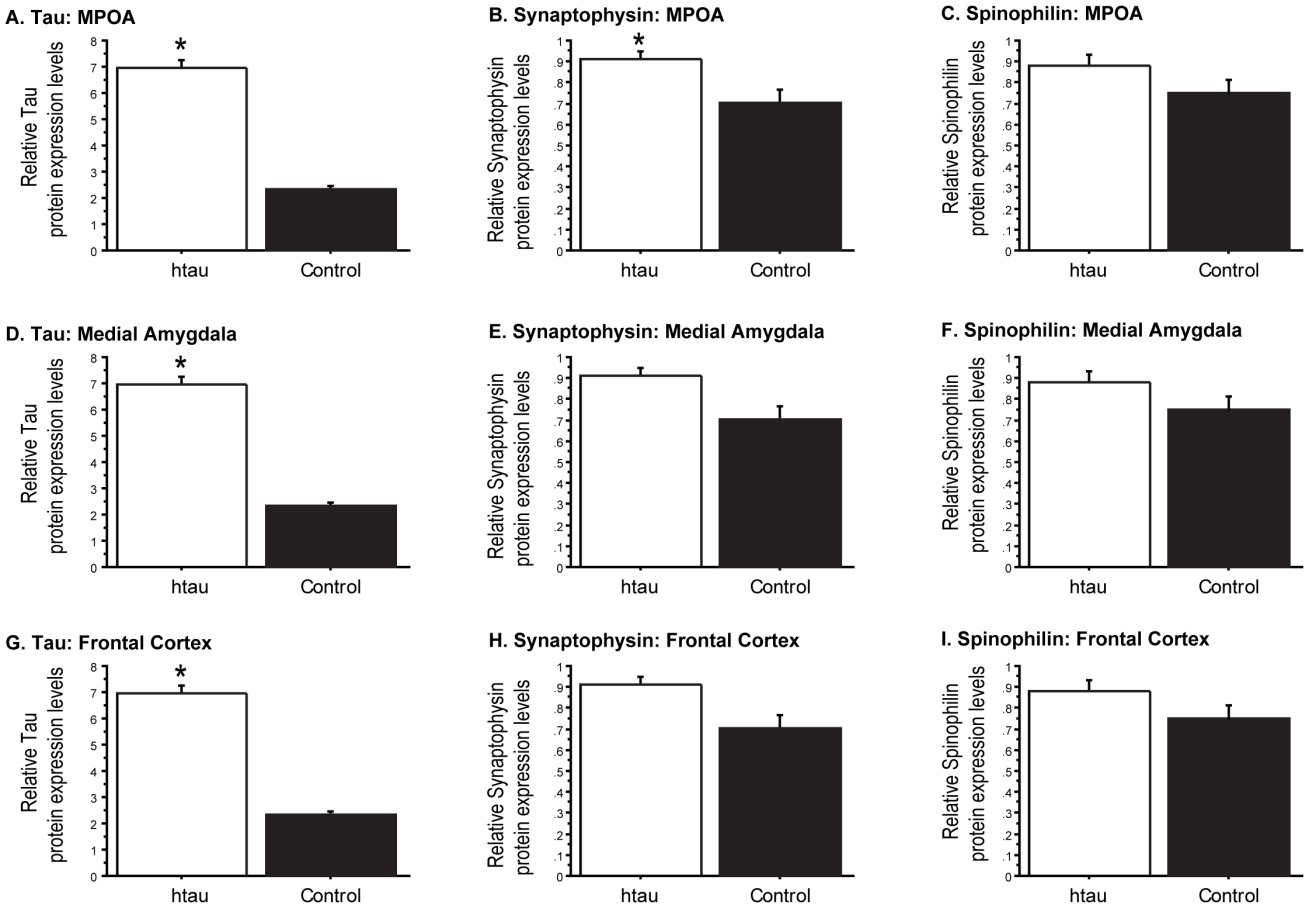
Tau, synaptophysin, and spinophilin protein expression levels in tau overexpressors and littermate controls. Tau, synaptophysin, and spinophilin protein expression levels in the (A-C) MPOA, (D-F) medial amygdala, and frontal cortex (G-I) of tau overexpressing mice and littermate controls 12 weeks after orchidectomy. (*p<0.05; levels normalized with an endogenous control, β-actin).

Tau levels in the MPOA of tau overexpressors were verified to be almost three-fold higher when compared to littermate controls (F_(1,22)_ = 222.151, p<0.05; Figure 4A). Tau was also elevated in the frontal cortex and medial amygdala (*p*<0.05 in all comparisons; Figure 4D & 4G). Synaptophysin levels in the MPOA of tau overexpressors were significantly higher than levels observed in controls (*p*>0.05, Figure 4B). A trend for higher spinophilin in the tau overexpressing mice vs. controls was noted in the MPOA (Figure 4C). Additionally, although both synaptophysin and spinophilin levels in the frontal cortex and medial amygdala were higher on average in the tau overexpressors than littermate controls, the differences were not statistically significant between the two groups (p>0.05; Figures 4E, F, H, & I).

### Experiment 3: Dendritic Architecture of MPOA Neurons Between B6D2F1 Hybrid Maters and Non-maters

**Figure 5:**
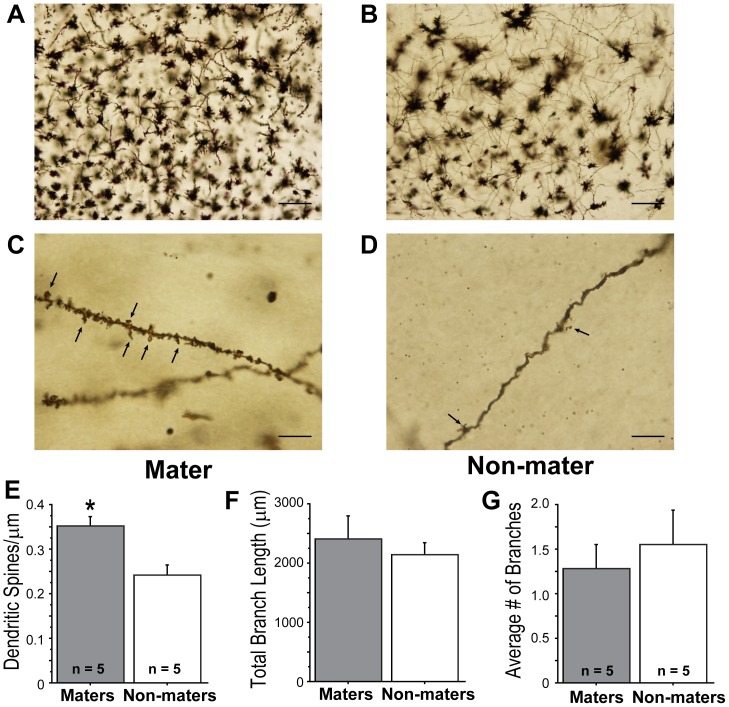
Maters have greater dendritic spine density than non-maters. Representative photomicrographs of Golgi-impregnated MPOA neurons from (A) a mater and (B) a non-mater (10× magnification; Scale bar, 100 mm). (C) Representative photographs of a dendritic segment from (C) a mater and (D) a non-mater (100× magnification; Scale bar, 10 mm). Arrows indicate dendritic spines. (E) Maters had increased dendritic spine density. There were no differences in (F) total dendritic length or (G) dendritic branching of MPOA neurons between maters and non-maters. Bars represent +SEM, *Significantly higher than non-maters (P<0.05).

Synaptophysin is found ubiquitously in synapses, and spinophilin is implicated in regulating spine formation and function [21]–[23]. Golgi-impregnated brains of orchidectomized B6D2F1 males were examined to assess dendritic morphology. We found that MPOA neurons in the maters had higher dendritic spine density than those found in non-maters (F_(1,8)_ = 13.313, p<0.05; Figure 5E). There were no differences in total dendritic length (F_(1,8)_ = 0.031, p>0.05; Figure 5F) or in the number of branch points (F_(1,8)_ = 0.326, p>0.05; Figure 5G) of MPOA neurons between maters and non-maters.

## Discussion

The results of our study demonstrate that tau may have a functional role in the expression of sexual behavior in the absence of gonadal steroids and perhaps the morphological differences we noted in neurons of the MPOA. Gonadal steroids, both androgens and estrogens, have a profound impact on dendritic morphology and patterns of synaptic connectivity in the adult central nervous system (reviewed in [24]). This is evident not only in brain areas classically associated with learning and memory, but in brain areas involved in the control of sexually dimorphic behaviors [25]–[27]. The results of these studies lend support to the hypothesis that MSB may be regulated by gonadal steroids in adulthood by promoting synaptic remodeling. However, unlike any of these studies in which the neuronal modifications were dependent upon steroidal action, the increased number of dendritic spines evidenced in the maters in our study correlate with striking differences in behavior independent of gonadal steroids.

Our characterization of the dendritic architecture between maters and non-maters in MPOA neurons are similar to results from previous studies that have investigated changes in dendritic morphology in the dorsal CA1 region of the hippocampus, albeit in response to gonadal hormones. These studies reported that hormone-induced changes in spine density reflect differences in spine number, rather than changes in dendritic length or branching [28] or axodendritic synapse density [29]. Notably, steroid replacement treatment (with either testosterone propionate or estradiol benzoate) is sufficient to restore MSB in non-maters [30]. It remains to be determined whether administration of exogenous steroids is sufficient to induce spinogenesis in non-maters. In light of the well-characterized ability of estradiol to regulate plasticity by affecting dendritic structure, particularly spinogenesis, in the neural circuitry underlying female sexual behavior, it is likely that gonadal steroids may regulate MSB through similar mechanisms [31].

Several lines of evidence now indicate that increased tau levels are functionally associated with steroid-independent MSB. Tau levels in the MPOA were higher in hybrid maters relative to non-maters, confirming the results of earlier gene expression data showing elevated *MAPT* expression in the MPOA of maters relative to non-maters [9]. Similar to the APP overexpressing mice, facilitation of mounting or intromitting behavior in a significant proportion of the tau overexpressors persisted for two months after orchidectomy relative to littermate controls. They did not display ejaculations beginning 10 weeks after orchidectomy, whereas a high percentage of orchidectomized B6D2F1 hybrid male mice were demonstrating this behavior well beyond 10 weeks after orchidectomy in previous reports [5], [7]. The relative decreased proportion of mice displaying ejaculations observed in the tau overexpressors may be caused by genetic background, as they were bred onto the C57BL/6J inbred-strain which normally does not demonstrate steroid-independent MSB. Additionally, it is likely that MSB is maintained by a combination of genes, and here we only manipulated one, *MAPT.* In addition to *MAPT*, there were three other microtubule-associated protein genes from the microarray analyses that were differentially expressed between B6D2F1 hybrid maters and non-maters. Similar to *MAPT*, microtubule-associated protein RP/EB family, member 3 (*MAPRE3*), microtubule-associated protein 4 (*MTAP4*), and microtubule-associated protein 1 A (*MTAP1a*) were more highly expressed in the MPOA of maters relative to non-maters. Further studies probing the role of these microtubule-associated proteins in steroid-independent MSB may provide further insight into the relationship between dendritic morphology and MSB. In addition to the tau overexpressing mice used in this study, there are several other transgenic mouse lines that overexpress tau [32]–[36]. However, none of these other lines have been closely examined for MSB prior to or after orchidectomy. One concern when studying behavior in adult tau overexpressors is the progressive accumulation of tau which then aggregates into neurofibrillary tangles leading to neurodegeneration which is normally found to start by ∼12 months of age [32]. The absence of steroid-independent MSB observed in our tau overexpressing mice 3 months after orchidectomy was unlikely related to neurodegeneration because at the termination of this study, the mice were ∼6 months of age. Cognitive impairments are also not likely to play a factor as these impairments begin to manifest at ∼9 months of age when hyperphosphorylated tau starts to accumulate [32], [37].

Abnormal filamentous tau deposits are considered a pathological characteristic in several neurodegenerative diseases (reviewed in [38]). However, in its non-pathological state, tau is implicated in cell survival, neuroprotection, supporting synaptic integrity and in facilitating cognitive behavior [39]–[44]. Prior to the onset of behavioral impairments in tau overexpressing mice that begin at ∼6–9 months of age, facilitated cognitive function as well as improved motor function were reported, demonstrating that tau plays an advantageous role in normal cognition and coordination prior to the accumulation of neurofibrillary tangles [37], [45], [46]. Thus, the elevated levels of tau found in the MPOA of hybrid maters and in the 2–6 month-old tau overexpressors we studied may play a beneficial role, particularly in synaptic integrity. This is supported by our finding that the B6D2F1 hybrid maters had higher levels of synaptophysin and spinophilin and that the tau overexpressors had higher levels of synaptophysin, but not spinophilin, in the MPOA. Additionally, higher expression levels of tau, synaptophysin and spinophilin were also found in B6D2F1 hybrid maters relative to non-maters in the medial amygdala, another area integral for MSB. In contrast, there were no differences in synaptophysin and spinophilin levels in the medial amygdala between tau overexpressing mice and their littermate controls. Overall, these results seem to indicate the potential existence of other molecular determinants that may control the expression of synaptic proteins associated with MSB. Further studies are required to determine the functional consequences of the increased levels of synaptophysin and spinophilin in steroid-independent MSB.

Interestingly, spinophilin is integral in establishing a signaling complex for dopaminergic neurotransmission through dopamine type-2 receptors by linking receptors to downstream signaling molecules and the actin cytoskeleton [47]. The relationship between dopamine and MSB has been well characterized in rodents (reviewed in [1]). Although the gene for the dopamine type-2 receptor was not differentially expressed between maters and non-maters in the microarray study, there is other evidence to suggest that dopamine may play a significant role in steroid-independent MSB. Sexually experienced orchidectomized rats, administered the dopamine agonist apomorphine, show partially restored MSB [48], [49] and estrogen receptor-α knockout mice, which usually show little MSB, copulated normally after apomorphine administration [50]. The exact relationship between spinophilin, dopamine, and steroid-independent MSB has yet to be determined and warrants investigation.

Reproductive behavior is a complex enterprise that not only includes genetic, molecular and neuroendocrine components, but is a pattern of specific behaviors that must be learned and consolidated (reviewed in [51]). In turn, copulatory behavior is subject to experience and requires a brain capable of experience-based synaptic plasticity. It seems unlikely that the increased dendritic complexity and elevated levels of tau, synaptophysin and spinophilin in the MPOA found in the maters relative to the non-maters were present prior to orchidectomy, as both groups received equivalent sexual experience. After orchidectomy, the maters had gained significantly more sexual experience than the non-maters. Because we did not directly compare the brains of the maters with sexually naïve mice, it is unknown whether the differences in dendritic morphology of MPOA neurons between maters and non-maters arise as a consequence of sexual experience. However, this seems unlikely given that sexual experience prior to orchidectomy and weekly behavioral testing after orchidectomy are not necessary for the expression of steroid-independent MSB in B6D2F1 hybrid males [6]. Moreover, increased dendritic complexity was not evident in the MPOA of sexually experienced rats relative to those that were sexually naïve [52].

Increased levels of tau (in its non-pathological state) and increased complexity of dendritic morphology have been associated with facilitated cognitive behavior [45], [46] (reviewed in [53]). Recently, studies in rats have shown that changes in dendritic morphology in areas classically associated with learning and memory and reward are associated with male sexual experience [52], [54]. These structural changes suggest that the learned components of MSB that are stored in memory are correlated with neuronal plasticity. However, because these brain areas as well as the male circuitry underlying MSB display a large capacity for hormone-driven structural plasticity, the role of learning per se is difficult to assess. The behavioral phenotypes demonstrated by orchidectomized hybrids allows for the investigation of the potential relationships between MSB and neuroplasticity independent of steroids. It remains to be determined if the decreased dendritic complexity found in the hybrid non-maters influences sexual behavior “memories,” leading to the inability of these males to maintain MSB. It is also unknown whether neurofibrillary tangles develop in the MPOA of the transgenic tau over-expressing mice and whether these tangles have functional consequences on steroid-independent MSB.

In summary, to our knowledge, our study is the first to make the link between increased dendritic complexity and increased levels of tau, synaptophysin, and spinophilin with steroid-independent MSB. How the differential expression of these proteins interact with each other and how they influence steroid-independent MSB has yet to be fully determined, but given our observation of increased dendritic spine density of MPOA neurons in maters relative to non-maters, we speculate that structural differences in synaptic morphology are involved. However, further studies investigating the exact relationship between increased levels of tau and relative increased dendritic spine density are necessary before a causal link can be established. Given declining libido during ageing in men, these studies could lead to discoveries with translational implications.
